# A Bio-Psycho-Social Co-created Intervention for Young Adults With Multiple Sclerosis (ESPRIMO): Rationale and Study Protocol for a Feasibility Study

**DOI:** 10.3389/fpsyg.2021.598726

**Published:** 2021-02-23

**Authors:** Valeria Donisi, Alberto Gajofatto, Maria Angela Mazzi, Francesca Gobbin, Isolde Martina Busch, Annamaria Ghellere, Alina Klonova, Doriana Rudi, Francesca Vitali, Federico Schena, Lidia Del Piccolo, Michela Rimondini

**Affiliations:** ^1^Section of Clinical Psychology, Department of Neurosciences, Biomedicine and Movement Sciences, University of Verona, Verona, Italy; ^2^Section of Neurology, Department of Neurosciences, Biomedicine and Movement Sciences, University of Verona, Verona, Italy; ^3^Section of Movement Sciences, Department of Neurosciences, Biomedicine and Movement Sciences, University of Verona, Verona, Italy; ^4^Latvian Academy of Sport Education, Riga, Latvia

**Keywords:** resilience, multiple sclerosis, health-related quality of life, well-being, physical activity, psychological intervention, patient engagement

## Abstract

**Background:**

Multiple sclerosis (MS), the most common neurological disease that causes disability in youth, does not only affect physical functions but is also associated with cognitive impairment, fatigue, depression, and anxiety and can significantly impact health-related quality of life (HRQoL). Since MS is generally diagnosed at a young age—a period of great significance for personal, relational, and professional development—adaptation can become highly challenging. Therefore, enhancing the competence of young people to adaptively cope with these potential challenges is of utmost importance in order to promote their potentialities and talents. It has been shown that psychological interventions targeting MS patients can enhance resilience and HRQoL and that regular physical activity (PA) and social engagement can improve psychological well-being. However, literature on the development of global interventions based on the bio-psycho-social model of the disease is missing. Even less attention has been paid to interventions dedicated to young adults with MS (YawMS) and to the involvement of patients in the development of such programs.

**Aims:**

In collaboration with MS patients, this study aims to develop a bio-psycho-social intervention (ESPRIMO) for YawMS, aiming to improve their HRQoL and to explore its feasibility, acceptability, and effects.

**Methods:**

To tailor the intervention to the specific needs of YawMS, “patient engagement principles” will be adopted in the co-creation phase, performing a web survey and focus groups with patients and healthcare professionals. In the intervention phase, a pilot sample of 60 young adults with MS will be enrolled. The co-created intervention, composed of group sessions over a 12-week period, will cover psycho-social strategies and include physical activities. Adopting a longitudinal, pre–post evaluation design, self-report questionnaires measuring HRQoL and other bio-psycho-social features (e.g., resilience, well-being, mindfulness traits, self-efficacy, perceived social support, psychological symptoms, illness perception, committed action, fatigue, attitudes, subjective norms, perceived behavioral control, motivation, perception of autonomy support for PA, barriers and intentions to PA) will be administered, the quantity and quality of PA will be measured, and a questionnaire developed by the authors will be used to evaluate the feasibility and acceptability of the ESPRIMO intervention.

## Introduction

Being the most common neurological disease that causes disability in young adults (Koch-Henriksen and Sørensen, 2010; Dimitrov and Turner, 2014), multiple sclerosis (MS) interferes with physical functions, such as gait, vision, and sensory abilities, and is often associated with cognitive impairment, fatigue, symptoms of depression and anxiety (Grossman et al., 2010; Narayanan et al., 2015; Boeschoten et al., 2017; Rainone et al., 2017; Tobin, 2019), and reduced health-related quality of life (HRQoL) (Grossman et al., 2010; Pappalardo et al., 2017; Rainone et al., 2017). Being diagnosed with MS represents a major life event, forcing the patient to psychological adjustments, which are, in part, independent of the direct consequences of neurological symptoms and disability. The MS diagnosis also influences patients’ psychological development, considering that it usually happens in a phase of life in which one’s own cognitive, emotional, and social functioning is still developing. In addition, the long-term clinical course of MS is highly unpredictable at the individual-patient level. Therefore, providing young people effective strategies to maintain well-being in the period immediately following the diagnosis is a key priority not only to prevent emotional distress but also to promote their potentials and talents.

### MS During Young Adulthood

Young people, when growing into the new roles of adulthood, often face increasing responsibilities and difficult decisions affecting their future. Thus, these years are a unique period for professional and personal development and for establishing interpersonal relationships, with significant implications also for health management (Park et al., 2006).

MS is generally detected during young adulthood, between the ages of 20 and 40. Approximately 85% of patients suffer from the relapsing–remitting form of the disease (RRMS), characterized by an unpredictable onset of neurological symptoms, potentially affecting the motor, somatosensory, visual, and other systems, which may recover completely or partially. This clinical form is related to a high degree of uncertainty not only in terms of frequency, clinical features, and severity of attacks but also in terms of the likely transition to a secondary progressive phase of the disease in up to 80% of cases. Aside from RRMS, 15% of MS patients present the primary progressive (PPMS) form in which disability accumulation is already apparent from the diagnosis. In 2006, Confavreux and Vukusic (2006) collected data from an observational study suggesting an association between age and clinical phenotype and disease course, namely, the age of patients with RRMS at onset was lower than those of patients with PPMS, however, falling below 40 years for the majority of subjects in both cases (median age 28.7 years for RRMS vs 39.1 for PPMS). Cognitive functioning in young adults with MS (YawMS) can also be influenced by the time of disease onset since it has been demonstrated that cognitive impairment tends to be prominent in older patients with long-standing disease (Ouellette et al., 2018; Brochet and Ruet, 2019). However, deficits of cognition can be detected also in young patients with MS, even with onset in the pediatric age (Banwell and Anderson, 2005; MacAllister et al., 2005; McKay et al., 2019).

Receiving a diagnosis of MS during this period of life makes acceptance of this chronic disease particularly challenging and makes psychosocial adjustment to the disease problematic, especially in the first years after the diagnosis (Kern et al., 2009; Moss-Morris et al., 2013; Multiple Sclerosis International Federation, 2013; Pagnini et al., 2014; Rainone et al., 2017; Strober, 2018; Gajofatto et al., 2019). For instance, a recent systematic review and meta-analysis (Rintala et al., 2019) suggests that patients with clinically isolated syndrome (which is the initial demyelinating event in most MS cases) or a recent MS diagnosis can suffer from mild to moderate symptoms of depression and anxiety. Among 18- to 30-year-old patients with MS, psychological distress resulted higher than in the general population, in particular, for men (Calandri et al., 2019). Social dimensions such as work, education, and interpersonal relationship are also involved in the process of adaptation to MS, where a sense of coherence has to be recreated (Thomas et al., 2006; Kiropoulos et al., 2016; Calandri et al., 2017). For the majority of patients, MS does not interfere with building new relationships; however, patients often feel embarrassed in public or worry about the impact of MS on their personal and work lives, including their plans to have children (Buchanan et al., 2010). In a study by Grytten and Måseide (2006), patients reported that their symptoms are often ignored or overemphasized in interpersonal encounters with healthy persons, thus making them feel “more ill” as when they stay alone. These results underline that the stigma of MS may lead to isolation and reduced social support and should thus be addressed in interventions also considering the implications for the individual and the community.

Considering these aspects, helping YawMS to develop adaptive skills represents a priority. Indeed it has been suggested that enhancing protective factors and resilient adaptation may contribute to a higher HRQoL of patients with MS (Kasser and Zia, 2020). Nevertheless, research dedicated to YawMS is still scarce, and better knowledge on how to promote well-being and HRQoL in this specific population is needed (Rainone et al., 2017; Silverman et al., 2017; Calandri et al., 2019).

### Adopting a Bio-Psycho-Social Approach in Promoting Quality of Life in MS

QoL, a multi-dimensional construct, is based on the principle that “health is a state of complete physical, mental, and social well-being and not merely the absence of disease or infirmity” (International Health Conference, 2002). HRQoL has a somewhat narrower focus, described as the impact of health, disease, and/or treatment on patients’ personal perception of their health status and functioning level as well as on their subjective well-being and satisfaction (Patrick and Erickson, 1993; Patti and Pappalardo, 2010; Megari, 2013; Pappalardo et al., 2017). In the clinical context, the evaluation of the multidimensional aspects included in the HRQoL subjective evaluation allows having a better understanding of patient perspectives and facilitates shared decision-making (Solari, 2005; Patti and Pappalardo, 2010; Yalachkov et al., 2019).

MS can have a severe impact on patients’ HRQoL which may be linked to disease-related (e.g., progressive course of disease, fatigue, cognitive impairment) as well as psychological (e.g., psychological symptoms, self-efficacy, coping strategies) and social factors (e.g., social support) (Strober, 2018). Patients react differently to adverse and stressful experiences, such as the diagnosis of a chronic disease or the onset of new symptoms. Several internal and external factors (e.g., coping strategies, personality traits, social support) might protect HRQoL and well-being from the negative impact of such experiences (Eiser and Lawford, 2009). Indeed how well patients with MS eventually adjust to the diagnosis and maintain their HRQoL seems to be linked to their resilience (Tan-Kristanto and Kiropoulos, 2015; Battalio et al., 2017; Rainone et al., 2017; Silverman et al., 2017; Kasser and Zia, 2020). Silverman et al. (2017) identified several bio-psycho-social factors, such as social support, planning, and physical wellness, which facilitate resilience in patients with MS (Silverman et al., 2017). Similarly, an observational study has recently started, with the aim to explore the role of disability-specific biological, psychological, and social factors on the resilience and HRQoL of YawMS (Gajofatto et al., 2019). In short, in the context of MS, it is highly important to adopt a bio-psycho-social approach when exploring and promoting HRQoL.

Considering these aspects, the multifaceted nature of resilience and HRQoL should also be considered in the development of interventions dedicated to patients with MS. However, the literature suggests that the existing interventions for well-being and HRQoL mostly focus on a single dimension (i.e., psychological interventions, physical activity (PA), or socialization programs). Despite the positive impact of interventions focusing on either physical, psychological, or social/interpersonal dimensions, there has been little discussion about the development of comprehensive interventions based on the bio-psycho-social model of the disease (Engel, 1977; McGuire et al., 2015), which might benefit from an integrated approach that takes into account the potential interconnections between biological, psychological, and social factors. Following this line of reasoning in the context of MS, psychological interventions for people with MS do not only have a positive effect on psychological aspects (Moss-Morris et al., 2013; Alschuler et al., 2018; Leclaire et al., 2018; Pakenham et al., 2018) but, investigating the link between mind and body, also on physiological outcomes (e.g., fatigue, physical vitality, sleep disturbances, pain) and on the perception of general health (Pagnini et al., 2014). Conversely, psychological well-being and HRQoL are enhanced by regular PA, which presents the additional advantage of reducing some symptoms of MS. A number of potential benefits have been reported in several reviews (Rietberg et al., 2004; Garrett and Coote, 2009; Andreasen et al., 2011; Motl and Pilutti, 2012; Latimer-Cheung et al., 2013; Motl, 2014) and meta-analyses (Motl and Gosney, 2008; Snook and Motl, 2009; Paltamaa et al., 2012; Pilutti et al., 2013; Asano and Finlayson, 2014; Ensari et al., 2014) for patients with mild or moderate MS performing PA, thus indicating that PA enhances health-related fitness and reduces impairment (Latimer-Cheung et al., 2013), fatigue symptoms (Andreasen et al., 2011; Pilutti et al., 2013; Asano and Finlayson, 2014), and depression (Ensari et al., 2014), strengthens balance (Paltamaa et al., 2012), reinforces walking function (Snook and Motl, 2009), and increases QoL (Motl and Gosney, 2008). To sum up, these exemplary interventions specifically targeting psychological and/or physical outcomes might be beneficial in all the domains of HRQoL. As regards the social domain, socialization and interpersonal relationships are aspects significantly influencing patients’ psychological well-being and illness perception. Moreover, social capital and social support have been shown to be associated with the physical and psychological impact of MS (Koutsogeorgou et al., 2019; Reyes et al., 2020).

Interconnection between bio-psycho-social domains might be evident not only in terms of outcome but also at process level (e.g., participation in intervention). For instance, considering the social domain, the effect of joy, socializing, and fun on people’s commitment to practice regular PA (Wiersma, 2001) has been widely recognized (El-Sherif, 2016). Indeed the opportunity to share the experience with other people and to create social connections can increase the participation rate (Bragg et al., 2009). In recent years, a number of prominent psychological approaches [for a meta-analytic review, see Hagger et al. (2002)] have been used to better understand the factors associated with patients’ participation in PA settings (e.g., projects aimed at promoting an active lifestyle, enhancing daily PA, stimulating the participation in exercise programs). Among them, the integrated behavior change (IBC) model for PA (Hagger and Chatzisarantis, 2014) has received widespread attention and has been successfully applied to patients. Indeed this model incorporates the very latest thinking on the psychological influences on behavior change and applies it to PA behavior. Many theories and models applied to PA behavior have intention or motivation as the focal construct, but the IBC model proposed an integration of these models. Adopting the IBC model as theoretical background in the assessment will allow exploring the volitional process of MS patients and the processes by which intentions are converted into behaviors to determine potential psycho-social factors influencing PA. In the MS field, patients with MS seem to be less active than healthy people (Motl, 2014). Some of the main reasons reported in the literature for MS patients’ lower activity are fatigue, impairment, and lack of time (Asano et al., 2013). Doing exercise in groups, however, has been evaluated as positive, motivating, and supporting (Clarke and Coote, 2015). Nevertheless, a study exploring all factors included in the IBC model in a group of young adults with MS has not been conducted yet. Therefore, exploring to which extent psychological (e.g., resilience, self-efficacy, perceived behavioral control) and situational (e.g., social support, perception of autonomy support) dimensions may impact the intention of young adults with MS to practice PA is crucial for promoting well-being and HrQoL.

### Patient Engagement as the Pathway for Grounding and Fostering the Quality of Interventions Designed for People With MS

Considering patients as partners in research is rapidly becoming a central priority for researchers and policy-makers as outlined by Bensing et al. (2013). During the last few years, the topic of patients’ involvement in chronic illness management has become increasingly important. The literature emphasizes the importance of promoting patient engagement through patient involvement, even in the planning and realization of such specific interventions addressed to them. Patient engagement also implies the development of interventions which are adapted to patients’ real needs and wishes and focused on patients’ daily challenges (Arafah et al., 2017). Nevertheless, scarce attention has been paid to the engagement of patients in the creation of such programs (Bensing et al., 2013).

Patient involvement in health research relies on the model of participatory research, which “seeks to engage communities throughout the research process on topics of practical relevance to those communities” (Rose, 2014). Participatory design is seen as a way to improve “the translation of clinical science into meaningful treatment” (Hofmann et al., 2015). In the context of MS, there has been an increasing interest toward patient engagement through shared decision-making and inclusion of the patients’ perspective (Yeandle et al., 2018). Considering patients’ experiences and concerns is essential for developing tailored and feasible interventions and for evaluating the process of change and improving quality of care. Increasing the use of these so-called patient-reported outcome measures in the MS field, including outcomes linked to a patient’s QoL, is of great relevance for patient engagement (Solari et al., 2010; Rieckmann et al., 2015). Moreover, qualitative methods are particularly useful for considering the patients’ perspective (Dennison et al., 2009). The relevance of involving patients with MS in tailoring their care has been recognized also in the Italian context (Ponzio et al., 2015), where participatory approaches have been used (e.g., Giovannetti et al., 2017, 2020).

### Aims

Drawing upon the different but interconnected strands of research described above, this study seeks to develop—in collaboration with patients with MS—a bio-psycho-social intervention (i.e., ESPRIMO intervention) for YawMS aimed at improving patients’ HRQoL. Furthermore, the study seeks to preliminarily test the feasibility of the ESPRIMO intervention and its effects in improving HRQoL using a pilot sample of YawMS.

This paper describes the rationale and study protocol of the ESPRIMO study.

## Methods and Analysis

### The ESPRIMO Study

**FIGURE 1:**
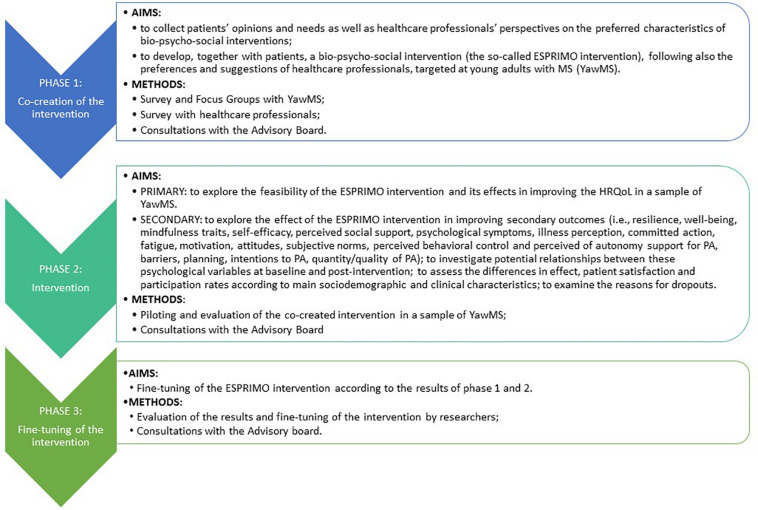
Graphical representation of the study design.

The ESPRIMO study will last 24 months, following three main consequential phases (Figure 1): phase 1 (co-creation of the intervention), phase 2 (intervention), and phase 3 (fine-tuning of the intervention).

The ESPRIMO study will be conducted during the COVID-19 pandemic. Thus, it seems unreasonable to start the co-creation of the intervention without taking into account the potential impact of this pandemic on the well-being of patients with MS and their care management. Therefore, in a preliminary explorative pre-phase, an online survey was conducted immediately after the end of the COVID-19 lockdown in Italy (May 2020), exploring the potential impact of the COVID-19 pandemic and changes in the management of care on the psychological needs of YawMS (Donisi et al., 2021). Indeed recent literature showed that patients with pre-existing vulnerabilities due to chronic disease experienced a high level of psychological distress as a consequence of the pandemic and of the changes in healthcare services (Akhoundi et al., 2020; Moghadasi, 2020; Umucu and Lee, 2020). Moreover, a recent study (Fitbit, 2020), using activity trackers, has shown that people were significantly less physically active during the COVID-19 lockdown. Thus, promoting well-being and PA in patients with MS during the COVID-19 pandemic is a priority.

### Participatory Design

A participatory design will be implemented by actively involving YawMS and healthcare professionals. An advisory board (AB) composed of YawMS and healthcare professionals will be established at the beginning and consulted throughout the project. The local section of the Italian MS Association will be invited to contribute in the recruitment and piloting activities.

### Phase 1: Co-creation of the Intervention

To tailor the intervention to the specific needs of YawMS, a co-creation phase will include data collection by administering surveys and performing focus groups. The main stakeholders, namely, patients and healthcare professionals, will be contacted. Results will be discussed with the AB and will be integrated in the theoretical framework of the intervention (see Table 1) to inform and define the contents and the characteristics of the intervention. Considering the pandemic phase, questions regarding YawMS’ views on e-health intervention will be also added to evaluate the option of telehealth modalities.

**TABLE 1:** Framework of the ESPRIMO intervention.

Bio component (physical activity)	Summary of the theoretical framework: Practical recommendations (Dalgas et al., 2009; Halabchi et al., 2017) and guidelines (Latimer-Cheung et al., 2013) for exercise and physical activity (PA) prescription for adult patients with multiple sclerosis (MS) will serve as an evidence-based framework to promote well-being and quality of life (QoL) through an active lifestyle. A systematic review by Pilutti et al. (2014) recommends PA as a safe intervention without side effects and adverse events for MS patients. Patients will be free to choose, according to their preferences and interests, between two PAs (i.e., dancing, walking), which have already been successfully used in patients with chronic illness and physical disability and which share common features, namely, they both represent pleasant activities, are performed in group sessions, and are characterized by a low level of activity intensity. The proposed exercises will then be adapted and tailored to the patients’ needs by experts in movement science and rehabilitation. For instance, a study by Samaei et al. (2016) investigating the effects of a 12-session walking group indicated that downhill walking on a treadmill enhances the patients’ balance control, muscle performance, and functional activity. Furthermore, dancing seems to be beneficial for the patients’ level of PA, gait, postural and balance control as well as emotional well-being and self-esteem (Salgado and de Paula Vasconcelos, 2010; Mandelbaum et al., 2016; Ng et al., 2020). There is evidence that low-intensity PA is associated with beneficial outcomes in MS patients (Motl and Gosney, 2008; Garrett and Coote, 2009). Motl et al. (2011) conducted a cross-sectional study that examined the association between free-living PA and neuropsychological measures of cognitive processing speed and learning and memory in persons with MS. PA was significantly correlated with cognitive processing speed, but not learning and memory, after controlling for sex, age, and education. Pleasantness is another important feature to consider as it stimulates motivation to participate in PA (Abele and Brehm, 1993; Biddle, 1995). Indeed it seems that walking is so popular because it is pleasant (Ekkekakis et al., 2008). Dancing programs, which combine cognitive demands and PA in a pleasant way, can serve as a non-pharmacological intervention for promoting mental and physical well-being in frail people (Zilidou et al., 2018).
	Main characteristics and contents: The participants will be involved in a PA program characterized by: - low level of activity intensity and exercises tailored to patients with MS; - pleasant activities to enhance motivation and evoke positive emotions. We selected low-intensity PA considering that patients with MS could have heterogeneous clinical deficits and different attitudes to PA. Experts in exercise and movement sciences and in adapted PAs will guide the intervention.
Psycho component	Summary of the theoretical framework: Main relevant psychological approaches in the context of MS will inform the intervention. Cognitive behavioral therapy has been shown to lead to reductions in depressive symptoms and anxiety, to an increase in adaptive coping strategies, and to an overall improvement of MS patients’ health-related quality of life (HRQoL) and well-being (Thomas et al., 2006; Moss-Morris et al., 2013; Hind et al., 2014; Fiest et al., 2016). Regarding the mind/body integration, the construct of mindfulness has received increasing attention in the field of chronic illness (Pagnini et al., 2015; Williams et al., 2015; Zhang et al., 2019) with positive effects of a mindfulness-based intervention on the HRQoL and fatigue and depressive symptoms in patients with MS (Grossman et al., 2010). The use of acceptance and commitment therapy (McCracken and Vowles, 2014; Feliu-Soler et al., 2018) has also shown promising results in patients with MS (Nordin and Rorsman, 2012; Pakenham et al., 2018; Giovannetti et al., 2020). Recently, positive psychology interventions, promoting positive constructs such as gratitude, optimism, and self-efficacy, have also been demonstrated to be beneficial for patients with MS (Müller et al., 2016; Anderson et al., 2017; Leclaire et al., 2018).
	Main characteristics and contents: The psychological component of the intervention will be administered in interactive group sessions to allow young adults with MS to share their experiences and socialize with each other. The general aim of the psychological group will be to improve the participants’ well-being, to enhance their resilience skills, to reduce stress perception, and to motivate to PA and social activities. Specific aims and contents will be decided according to the results of the survey and the focus groups. Two clinical psychologists with clinical experience in the MS field will guide the groups.
Social component	Summary of the theoretical framework: Group interventions have a long tradition in psychology and have been associated with several positive aspects, such as social support, hope, sense of belonging, universality, and interpersonal learning (Yalom, 1995). Shared experience has been considered by patients as a positive, non-specific factor in a recent psychological intervention for patients with MS (Giovannetti et al., 2020). In a qualitative study on engagement of patients with MS in PA, group membership contributed strongly to patients’ positive perceptions of PA (Clarke and Coote, 2015).
	Main characteristics and contents: The group-based PA and psychological activities (i.e., eight to 10 participants) will help to promote social belonging and foster relationships among participants. Social activities (e.g., dance evenings, social events, outdoor walking) involving patients with MS and the general population will be proposed in order to reduce stigma and exclusion and facilitate the creation of good relationships, the experience of positive emotions, and the sense of belonging.

#### Survey With YawMS

A cross-sectional web-based survey, using a self-administered anonymous questionnaire, will be implemented using the application LimeSurvey and promoted on social media (i.e., Facebook, Instagram). The survey will aim to explore YawMS’ opinions regarding the preferred contents and characteristics of a bio-psycho-social intervention and possible barriers/facilitators for participation (Supplementary Appendix 1: survey with YawMS). Socio-demographic and clinical data (e.g., age, gender, educational level, type of MS) will also be collected to evaluate potential differences across sub-groups of YawMS. The survey will be open for 2 months to reach a sample of at least 50 YawMS according to the following inclusion criteria: age 18–45 years, MS diagnosis, Italian speakers, and signed electronic informed consent.

#### Survey With Healthcare Providers

A cross-sectional web-based survey, using the application LimeSurvey, will be conducted on a cohort of healthcare professionals working with MS patients. The self-administered, anonymous questionnaire will aim to assess healthcare professionals’ opinions regarding the contents of a bio-psycho-social intervention and possible barriers/facilitators to patient participation (Supplementary Appendix 2: survey with healthcare professionals). The survey will also collect information on socio-demographic characteristics, role, and experiences of the participants. The survey invitation email will be sent to all healthcare professionals of the MS Hub and SPOKE network of Verona Province. Moreover, aiming to reach a sample of at least 25 respondents, the participating professionals will be asked to send the survey to two other colleagues. Considering the bio-psycho-social nature of the ESPRIMO intervention and the multidisciplinary nature of MS care, diverse healthcare professionals will be involved in the survey, mainly including neurologists, clinical psychologists, experts in rehabilitation, neuropsychologists, MS nurses, and physiotherapists. This will allow having a more comprehensive picture of YawMS’ needs, including the perspective of different healthcare professionals working with YawMS. The inclusion criteria are as follows: being a healthcare professional working with MS patients, Italian speakers, and signed electronic informed consent.

#### Focus Groups With YawMS

Focus groups involving YawMS will be conducted with the aim to deepen the understanding of the survey results. In particular, focus groups will provide additional results on preferences and needs in terms of psychological, physical, and socio-relational contents of the intervention and on potential strategies to reduce barriers to participation. Specifically, Supplementary Appendix 3 provides an overview of the topics that will be covered during the focus groups’ discussions. Focus groups will be conducted online and video recorded. The transcription will be analyzed by applying inductive content analysis. Furthermore, the AB will be consulted to assess the completeness and relevance of the results. Applying the criteria of data saturation (Onwuegbuzie et al., 2009), two focus groups, each composed of six to eight patients, are expected to be sufficient to explore all the relevant topics. Patients will be recruited in clinical centers and through MS associations, balanced by gender and clinical characteristics as much as possible, according to the following inclusion criteria: age 18–45 years, MS diagnosis, Italian speakers, and signed electronic informed consent. Before participating, the focus group discussants will fill out a brief socio-demographic and clinical questionnaire.

### Phase 2: Implementation of the Intervention

**FIGURE 2:**
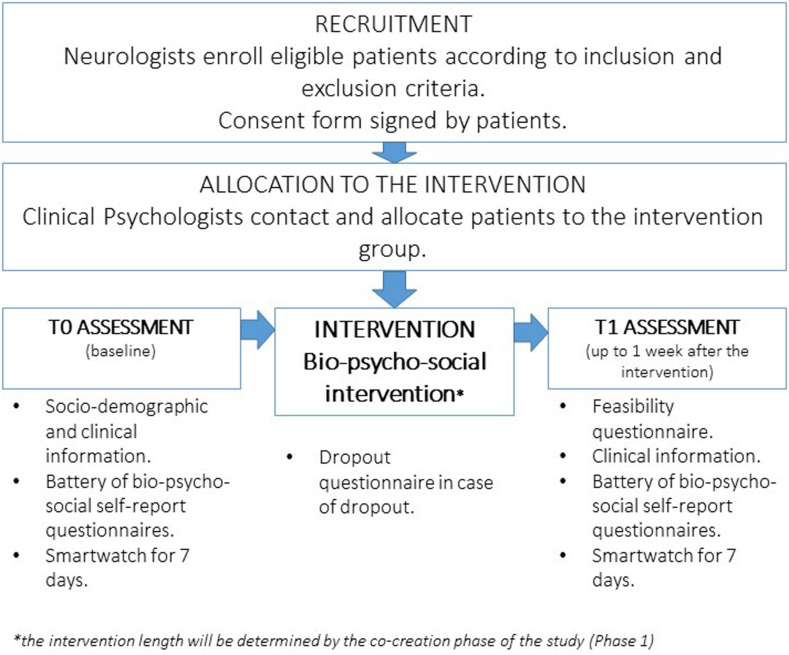
Implementation of the intervention procedures.

The intervention phase will follow the process described in Figure 2. Patients will be enrolled according to the following inclusion criteria: age range 18–45 years, MS diagnosis as reported by the treating neurologist in the medical record according to the revised McDonald Criteria (Thompson et al., 2018), Italian speakers, and signed informed consent. Patients with the following characteristics will be excluded: clinically relevant cognitive deficits (as evaluated by the treating neurologist) which may represent obstacles in filling the questionnaires and the intervention participation; severe psychiatric disorders, such as psychosis, bipolar disorder, active substance abuse problems, dissociative disorders, or a current diagnosis of major depression (as evaluated by the treating neurologist or the clinical psychologist on the basis of the medical record); and clinically relevant physical impairments rendering impossible the PA intervention, defined as an Expanded Disability Status Scale (EDSS) score higher than 3.5 (Bowen et al., 2001). Considering the lack of a clear age cutoff for the definition of “young adult” in the literature, the age range 18–45 years was defined in the current study, consistent with previous research in the medical field (Garcia and Finlayson, 2005; Pezzini, 2012; Andersson and Vasan, 2018), representing a slightly widened age range of MS disease onset (i.e., 20–40 years). Eligible patients can start the baseline assessment phase and the subsequent intervention at least 3 months after a relapse and if disease-modifying therapy has been stable for at least 1 month.

Eligible patients will be enrolled by the neurologists/residents working at the MS Center of Borgo Roma Hospital in Verona (MS Hub Center, northeast of Italy). Moreover, neurologists of affiliated SPOKE clinics will refer potentially eligible patients to the Hub Center of Borgo Roma. During the patients’ first visit at the MS center, the neurologists/residents will explain the study and assess the fulfillment of inclusion criteria/the absence of exclusion criteria. Those willing to participate will sign a consent form stating that they will be contacted by the clinical psychologist of the Clinical Psychology Unit, Borgo Roma Hospital in Verona, in charge of the piloting phase regarding the allocation to the available intervention session and the schedule of the assessment phase (T0–baseline). Considering that the intervention will be realized as a group intervention, the intervention will start as soon as eight to 10 YawMS will have been enrolled. The patients will then be contacted when the subsequent group starts.

#### Socio-Demographic Variables and Bio-Psycho-Social Questionnaires

Socio-demographic and clinical characteristics (e.g., age, gender, marital status, educational level, employment, living situation, MS type, months since diagnosis, year of MS onset, MS treatment, receiving psychological and PA) will be collected at baseline (T0) using routine clinical and administrative forms and a questionnaire developed by the authors. At baseline (T0) and post-intervention (T1) up to 1 week after the intervention, the participants will fill out a battery of questionnaires (Table 2) to measure the ESPRIMO intervention effect. Moreover, at post-treatment (T1), we will record additional information, referring to the time during the intervention, to assess potentially confounding variables (i.e., stressful life events not related to MS, use of psychoactive drugs, receiving psychological interventions, level of physical activity and physical activity habits, MS relapses and symptoms).

**TABLE 2:** Battery of bio-psycho-social and clinical questionnaires.

Measure	Questionnaire^*a*^
Health-related quality of life (primary outcome)	The COOP/WONCA charts (van Weel et al., 1995; Pappalardo et al., 2017; Taghipour et al., 2018)
Resilience	The Connor Davidson Resilience Scale (CD-RISC 25) (Connor and Davidson, 2003; Senders et al., 2014; Black and Dorstyn, 2015; Battalio et al., 2017; Koelmel et al., 2017)
Mindfulness traits	Five Facet Mindfulness Questionnaire—Short Form (FFMQ-SF) (Baer et al., 2006; Giovannini et al., 2014; Hoogerwerf et al., 2017; Iani et al., 2017; Nauta et al., 2017; Hoogerwerf et al., 2017; Senders et al., 2018; Spitzer and Pakenham, 2018)
Self-efficacy in multiple sclerosis (MS)	The Self-Efficacy in Multiple Sclerosis Scale (SEMS) (Calandri et al., 2018, 2019; Bonino et al., 2018)
Perceived social support	Multidimensional Scale of Perceived Social Support (MSPSS) (Zimet et al., 1988; Prezza and Principato, 2002; Koelmel et al., 2017)
Anxiety and depression symptoms	Hospital Anxiety and Depression Scale (HADS) (Zigmond and Snaith, 1983; Costantini et al., 1999; Honarmand and Feinstein, 2009; Giordano et al., 2011)
Illness representations	Brief Illness Perception Questionnaire (Brief B-IPQ) (Broadbent et al., 2006, 2015; Pain et al., 2006; Moss-Morris et al., 2009; Carletto et al., 2017)
Well-being	Short Form 12 Health Survey (SF-12) (Nortvedt et al., 2000; Apolone et al., 2001)
Committed action	Committed Action Questionnaire (CAQ-8) (McCracken, 2013; McCracken et al., 2015)
Fatigue	Fatigue Scale for Motor and Cognitive Functions (FSMC) (Penner et al., 2009; Elbers et al., 2012)
Perceived autonomy support to physical activity (PA)	Perceived Autonomy Support Scale for Exercise Setting (PASSES) (Hagger et al., 2007)
Autonomous motivation to PA	The Behavioral Regulation in Exercise Questionnaire (BREQ-3) (Markland and Tobin, 2004)
Attitudes toward PA	Scale developed by Galli et al. (2018), following the recommendations of Ajzen (1991)
Subjective norms	Scale developed by Galli et al. (2018), following the recommendations of Ajzen (1991)
Perceived behavioral control	Scale developed by Galli et al. (2018), following the recommendations of Ajzen (1991)
Intention	Scale developed by Galli et al. (2018), following the recommendations of Ajzen (1991)
Planning	Scale developed by Galli et al. (2018), following the recommendations of Ajzen (1991)
Barriers and facilitators	Barriers to Physical Activity Questionnaire for People With Mobility Impairments (BPAQ-MI)—adapted (Vasudevan et al., 2015)

*^a^The Italian version/translation of the questionnaire will be used. References regarding the utilization of the questionnaire in the MS context are included when available.*

Moreover, a smartwatch Polar Vantage M will be used by each patient for 7 days (evaluating a complete week, also including the weekend) at baseline and 7 days after the intervention to compare the quality (i.e., intensity, typology of PA) and quantity (i.e., duration, frequency) of physical activities during each day. In addition, the Polar Vantage M will evaluate other physical parameters [e.g., number of steps/day, kilometers traveled/day, number of active hours/day, number of inactive hours/day, number of hours of sleep/day, heart rate (HR), heart rate variability, and estimated kilocalories consumed/day]: the rationale behind this evaluation is to measure all these objective parameters which will support us in measuring the effects of the PA intervention on MS patients’ well-being and HrQoL. The reliability of the Polar Vantage M smartwatch has been reported for measurements of physical activity and HR at different treadmill exercise intensities (Climstein et al., 2020). Moreover, a recent study measuring the physical activity and HR of preschoolers demonstrated the validity and social acceptability of the Polar Vantage XL smartwatch, which is very similar to the Polar Vantage M model (Bar-Or et al., 2020).

Furthermore, potential effect confounders (i.e., disability and neuro-psychological decay) will be considered and evaluated both at T0 and T1, namely, the neuropsychological aspects and the level of disability will be measured with the Symbol–Digit Modality Test (Smith, 1973) and the EDSS (Bowen et al., 2012), respectively.

#### Feasibility Assessment and Outcomes

After the intervention (T1), a questionnaire developed by the authors using close (rated by Likert scales) and open questions will be administered to evaluate the acceptance and satisfaction of the intervention. The aims of the feasibility assessment will be to explore pleasantness, utility, feasibility, opportunity, and risk for future development (Supplementary Appendix 4: feasibility questionnaire). The collected quantitative and qualitative information will help to adapt the intervention and its administration and reduce participation barriers for future MS patients.

As regards feasibility outcomes, the self-reported evaluation of feasibility of the study by participants on a Likert scale ranging from 1 (not at all) to 10 (very much), with higher scores reflecting higher levels of feasibility according to the participants, will represent the primary feasibility outcome of the intervention (i.e., *a priori* feasibility cutoff of 6 has been established). Moreover, the overall number of dropouts and the exact time of dropping out will be considered as secondary outcomes evaluating the feasibility of the intervention. Considering the MS disease characteristics and previous research with MS populations, we have hypothesized a feasibility dropout cutoff rate within the range 20–40%. Moreover, patients dropping out during the interventions will be contacted by a clinical psychologist of the research team not involved in administering the intervention. Using a specific questionnaire, the underlying reasons for dropping out will be examined.

#### Piloting of the Intervention

The intervention will last for approximately 10–12 weeks of PA and six to eight encounters of psychoeducational intervention of around 2 h, with the primary aim of improving the HRQoL of YawMS. The exact frequency of the intervention and the specific aims and contents of the intervention, together with the modalities (e.g., MS clinic setting, virtual setting), will be based on the results of the co-creation phase, which represents the key strength of the current study. Nevertheless, considering that, due to the current pandemic, several restrictions to social activities are in place to reduce the risk of contagion and that, since the start of the pandemic, telemedicine has gained increased diffusion and people got gradually used to these new ways of interaction, we expect that, in the co-creation phase, the participants will embrace these new modalities.

PA and psychological interventions will be implemented in the same period of time (i.e., from the allocation to the intervention group) to maximize the benefit of the intervention, but in separate and dedicated sessions. An ideal timeframe might be 12 weeks, where one psychological and one PA session are proposed in the first and last week, while in the central weeks, psychological sections could be provided bimonthly and PA sessions weekly.

### Intervention

The intervention will embrace three highly integrated major components (bio, psycho, and social components). Table 1 shows the preliminary theoretical framework of the intervention based on the literature in the respective fields. The approaches described in Table 1 will guide the intervention activities both in terms of contents and methods and will be adapted on the basis of the participants’ preferences expressed in the surveys and focus groups.

Psychological and PA interventions will be guided by an expert in group activity management. The group dimension of psychological and PA sessions will allow the participants to share their experiences, receive peer support, increase a sense of belonging, and promote relationships and socialization, which will benefit their overall well-being. Psychological and PA homework will be suggested to promote the application of new strategies and habits in everyday life.

The design of the study (i.e., a single-arm, pre-/post-intervention, within a pilot approach) does not require a formal power analysis to determine the sample size (Eldridge et al., 2016). However, using Coop/Wonca as a measure of HRQoL and considering the final Coop/Wonca chart (namely, “perceived overall health”), on the basis of previous literature on this instrument (van Weel et al., 1995; Moretti et al., 2012; Pappalardo et al., 2017) (power, 0.80; alpha, 5%; paired sample t-test; 30% dropout rate; the estimated effect size corresponds to a reduction of the mean scores of at least 0.5), a number of 43 patients with MS has been considered as sufficient for the intervention. To account for a different scenario regarding the dropout rate (range, 20–40%), we need to enroll between 54 and 72 patients. Therefore, we aim to recruit about 60 participants. Patients will be considered as dropouts if their attendance to the psychological and physical sessions is lower than 75%.

### Data Analysis

The survey results will be analyzed using descriptive statistics. An inductive content analysis will be applied to analyze the focus group discussions. This methodology, where categories are derived directly and inductively from the raw data, allows researchers to immerse themselves in the narrative flow of the participants’ discussion without being led by preconceived theoretical perspectives (Moretti et al., 2011). All focus groups will be videotaped and the discussions transcribed. The participants’ opinions will be reported in an Excel file, and two researchers (SP and VD), in line with the guidelines in the literature (Moretti et al., 2011), will analyze the text and elaborate possible labels. These labels will then be compared in a plenary meeting, and concordant and discordant labels will be discussed with a third reviewer (MR). As a final step, all answers will be then coded using the finalized labels, and the frequency distribution will be calculated for each category. Regarding the information gathered in phase 2, the feasibility questionnaire will be summarized using a descriptive approach. The effects of the intervention on outcomes, evaluated in terms of a pre–post design, will be explored to assess the changes of the individual scores using paired data techniques (correlations and hypothesis tests, non-parametric or parametric where appropriate). All analyses will be performed using STATA 15.1 (2020).

## Discussion

As stated in the introduction, considering the challenges imposed by MS in the delicate phase of young adulthood, planning care and early interventions for this age group is of utmost importance for their well-being and for reaching their potentials. Such support strategies may positively affect the evolution and the perception of neurological symptomatology and improve the process of MS acceptance and adaptation. To increase the ecological validity of intervention dedicated to YawMS, a thorough understanding of their preferences and expectations is needed. The ESPRIMO project will allow filling this gap using a participatory design throughout the different phases of the study. Online modalities for the co-creation methods have been preferred in the current protocol due to the restrictions linked to the current COVID-19 pandemic, thus guaranteeing the highest grade of safety and protection for the participants. While this methodology comes with the limitation that the fulfillment of the inclusion criteria was self-evaluated by participants, limiting the control on the sampling, we also considered some advantages of web-based surveys that fitted to the explorative nature of the co-creation study and/or to the specific needs of people with MS: (1) ability to reach a larger pool of potential participants within a shorter period of time, (2) access to a larger population (entire Italian territory), including patients who were difficult to reach, and (3) enhanced comfort and sense of control for the participants (Ahern, 2005; Menon and Muraleedharan, 2020). The low cost and the efficiency of data collection and management are favorable elements as well.

For this reason, at the end of the study, it will be possible to offer a bio-psycho-social intervention to YawMS, tailored to their needs and aimed to foster their HRQoL. The participatory design of the study will also improve trust and collaboration among MS stakeholders as a potential starting point for future research aiming to improve MS quality of care. In line with this, the ESPRIMO project will promote the collaboration of a multidisciplinary team of researchers and healthcare professionals in the MS field. Therefore, the bio-psycho-social intervention will foster a holistic vision of MS care in the clinical practice.

ESPRIMO will adopt a bio-psycho-social approach, including highly integrated interventions in physical, psychological, and social domains, which will be administered in the same time period to maximize the benefits that the components have on each other. In the short term, we expect that fostering psycho-social strategies and promoting PA will help YawMS to better cope with distress linked to MS, enhance their HRQoL and general well-being, and positively affect the evolution and the perception of neurological symptomatology. In the medium term, the consolidation of these strategies will promote a growing sense of auto-efficacy. For example, the application of strategies for emotional and cognitive re-elaboration as well as for the acceptance of the limits imposed by MS will lead to a better adaptation to the health condition, treatment, and possible side effects. In the long term, we expect that the developed resilient attitude will help the patients also in other critical areas of their personal and social life, thus reducing distress and improving the patients’ global QoL.

Social support is considered a fundamental resource for QoL and well-being. For this reason, both psychological and physical interventions will be performed in group sessions to create a collaborative and supportive network among patients of similar age and with similar challenges and difficulties. Although an analysis of cost-effectiveness would exceed the scope of our project at this stage, it can generally be said that group treatments have also the advantage of reduced costs compared to individual interventions.

The IBC model (Hagger and Chatzisarantis, 2014) has received widespread support (Galli et al., 2018) and is one of the most frequently applied and tested in health behavior research and in the health domain in general and has been used for studying the PA effects on health in particular. Using the IBC model for PA, we aim to explore a set of personal and situational factors linked to the promotion of well-being and to enhance HRQoL through PA in YawMS, considering both intention and motivation as focal constructs. This aim is particularly original as the model and linked scales have not been previously used in the context of MS.

To conclude, we expect that the ESPRIMO intervention will be a promising and feasible approach in improving the HRQoL of YawMS based on their specific needs. The bio-psycho-social contents of the intervention will positively influence the physical, social, and emotional domains of HRQoL. Moreover, the integrated implementation of the bio-psycho-social components of the intervention should re-enforce the effect of each single component, creating a virtuous circle of resilient resources and positive experiences for YawMS. A randomized, controlled trial with follow-up on a larger sample could be used to test the efficacy as well as the cost-effectiveness of this approach in the future.

## Ethics and Dissemination

A dissemination plan will be established in the first phase of the project, targeting different stakeholders including patients, the scientific and clinical community, and the general population. Considering the young age of the participants, we will promote the project, in particular, on social media (e.g., online groups of YawMS on Facebook, Instagram). As previously discussed, social media is a popular means of interaction for adolescents and young adults, and healthcare providers have recognized its potential of engaging this specific population and disseminating tailored health education messages (Wong et al., 2014). We expect that the use of social media channels will increase patient engagement, may represent a further occasion to disseminate health messages, and will heighten the interest of patients in health initiatives (e.g., results of the co-creation phase on strategies to deal with MS). Considering the social dimension of the project, the use of social media might represent a starting point for other promising initiatives in the online community for YawMS. Having the local MS association involved in the project will allow translating the social activities and events into real life, thus increasing the patients’ sense of belonging and positive experiences.

Ethical approval for the entire study has been obtained from the Ethics Committee of the Verona and Rovigo Province (Prog. 2676CESC). The study has been registered at ClinicalTrials.gov (ClinicalTrials.gov ID: NCT04431323). The study will be conducted in compliance with the latest revision of the Helsinki Declaration as well as the Oviedo Declaration, thus guaranteeing strong ethical standards. The principal investigator (MR) will act in accordance with the responsibilities established by the rules of Good Clinical Practice (Legislative Decree 211/2003) and in compliance with the laws and regulations enforced on data protection, including the General Data Protection Regulation (EU) 2016/679 regarding personal data.

## Data Availability Statement

The data and materials used during the current study are available from the corresponding author (MR) on reasonable request.

## Ethics Statement

The studies involving human participants were reviewed and approved by Ethics Committee of the Verona and Rovigo Province (Prog. 2676CESC). Written informed consent was not provided because the paper presents the protocol of the study. However, participants will provide their informed consent to participate in the study.

## Author Contributions

VD, AGa, MM, MR, FS, and LD conceived and designed the study. VD, FV, AGa, MM, and MR were responsible for the collection of data. AGa, FG, and AGh recruited the participants. VD, FV, MR, DR, and AK were responsible for performing the intervention. VD, FV, AGa, MM, IB, and MR analyzed and interpreted the data. VD, AGa, AGh, FG, MM, IB, FV, and MR drafted the manuscript. All the authors read and approved the final manuscript.

## Conflict of Interest

The authors declare that the research was conducted in the absence of any commercial or financial relationships that could be construed as a potential conflict of interest.
