# Supplementary material for: A Bio-Psycho-Social Co-created Intervention for Young Adults With Multiple Sclerosis (ESPRIMO): Rationale and Study Protocol for a Feasibility Study
Source: Front Psychol. 2021 Feb 23;12:598726. doi: 10.3389/fpsyg.2021.598726 (PMC7940381; doi:10.3389/fpsyg.2021.598726)
Supplement: Supplementary file 1 [file Table_1.docx]

Supplementary Material

**Appendix 1 Survey with young adults with MS**

**SECTION A: SOCIO-DEMOGRAPHIC AND CLINICAL ASPECTS**

- Age: ____________

- Gender

- Male
- Female
- Other

- Relationship status:

- Single
- Married/ living with a partner
- Other

- Degree:

- No degree
- Middle School License
- High School Diploma
- Undergraduate/Graduate Degree

- Employment:

- Student
- Employed
- Looking for employment
- Other

- Diagnosis:

- Clinically isolated syndrome (CIS) or radiologically isolated syndrome (RIS)
- Relapsing-remitting multiple sclerosis (RRMS)
- Secondary progressive multiple sclerosis (SPMS)
- Primary progressive multiple sclerosis (PPMS)

- How much time has passed since the diagnosis?

- less than a year
- 1-2 years
- 2-5 years
- more than 5 years

- Interference of the disease with movements/energy:

Your health currently limits you in carrying out activities that require moderate physical effort, such as moving a table, using a vacuum cleaner or taking a ride on a bicycle.

- Yes, it limits me a lot
- Yes, it limits me partially
- No, it doesn’t limit me at all.

**SECTION B: ASPECTS RELATED TO YOUR NEEDS AND PREFERENCES**

**The psychophysical well-being of each of us is based on a healthy lifestyle. The following questions are aimed at understanding how to develop an integrated intervention that takes care of the body and mind at the same time.**

- In your opinion, how much can your lifestyle affect your disease?

*Not at all* 1 2 3 4 5 6 7 8 9 10 *Much*

- In your opinion, how important is it that an intervention is based on an integrated vision of mind and body?

*Not important* 1 2 3 4 5 6 7 8 9 10 *Very important*

- In your opinion, how important is it for an intervention to aim to improve psychological well-being and physical well-being at the same time?

*Not important* 1 2 3 4 5 6 7 8 9 10 *Very important*

- In your opinion, in order to promote PSYCHOLOGICAL well-being, what are the main objectives on which an intervention should focus (indicate a maximum of 4)?

- It should motivate me to change.
- It should change the way I see things.
- It should inform me about the risks of an unhealthy lifestyle.
- It should help me to accept the disease and its consequences.
- It should provide me with advice and stress management strategies.
- It should make me aware of my emotions.
- It should increase my sense of self-efficacy in managing the disease.
- It should help me to process past traumas.
- It should help me to express my emotions and concerns.

- Are there any other psychological aspects that you find useful for young people with MS?

- Yes
- No

- If so, which ones?

- In your opinion, what characteristics should the proposed physical activity have in order to promote PHYSICAL well-being (indicate a maximum of 2)?

- It should be fun.
- It should let me meet other people.
- It should be adapted to my physical needs.
- It should encourage me to continue with physical activity even after the intervention.
- It should have tangible benefits for the body.
- It should teach me something new.
- It should let me get to know new persons.
- It should be a new activity.

- Are there any other aspects of physical activity that you find useful for young people with MS?

- Yes
- No

- If so, which ones?

- In your opinion, what would be the ideal frequency for meetings:

- Two meetings per week
- One meeting per week
- One meeting every other week
- One meeting per month

- In your opinion, what would be the ideal venue for meetings related to psychological aspects:

- My hospital or treatment center
- The seat of a patient association
- A neutral place not connected to my illness (e.g., gym, social club)
- Via telematics (using a videoconferencing platform, e.g., Zoom, skype)

- In your opinion, what would be the ideal venue for meetings relate to physical activity:

- My hospital or treatment center
- The seat of a patient association
- A neutral place not connected to my illness (e.g., gym, social club)
- Via telematics (using a videoconferencing platform, e.g., Zoom, Skype)

- If the intervention would be delivered via telematics, what benefits would you see?

- If the intervention would be delivered via telematics, which critical issues would you see?

**SECTION C: SUGGESTIONS**

- In general, what could be a barrier for you to take part in these meetings?

- In general, which strategies could be used to overcome the barrier(s) you have listed?

**Appendix 2 Survey with healthcare professionals**

**SECTION A: SOCIO-DEMOGRAFIC AND WORK-RELATED ASPECTS**

- Age:

- Gender

- Male
- Female
- Other

- Profession:

- Neurologist
- Physiatrist
- Nurse
- Psychologist
- Physiotherapist
- Other ________________

- Years of working experience with patients with MS:

- Less than 1 year
- Between 1 and 5 years
- Between 5 and 10 years
- More than 10 years

**SECTION B: ASPECTS RELATED TO THE NEEDS AND PREFERENCES OF YOUR PATIENTS**

**The psychophysical well-being of each of us is based on a healthy lifestyle. The following questions are aimed at understanding how to develop an integrated intervention that takes care of patient’s body and mind at the same time. Please think about your young patients with MS (age 18-45):**

- In your opinion, how much can patients’ lifestyle affect their disease?

*Not at all* 1 2 3 4 5 6 7 8 9 10 *Much*

- In your opinion, how important is it that an intervention is based on an integrated vision of mind and body?

*Not important* 1 2 3 4 5 6 7 8 9 10 *Very important*

- In your opinion, how important is it that an intervention aims to improve psychological well-being and physical well-being at the same time?

*Not important* 1 2 3 4 5 6 7 8 9 10 *Very important*

- In your opinion, how much can an integrated biopsychosocial intervention be beneficial for the treatment process?

*Not at all* 1 2 3 4 5 6 7 8 9 10 *Much*

- If you have indicated a score of at least 3, which benefits could it have?

While responding to the following questions, we ask you to:

- refer to young patients with multiple sclerosis (age 18-45 years) with a level of disability at most moderate (Expanded Disability Status Scale (EDSS) score less than 3.5)

- do not dwell on considering the conditions and limitations related to the current pandemic but refer to the usual treatment process

- In your opinion, in order to promote the PSYCHOLOGICAL well-being of patients, which are the main objectives on which an intervention should focus (indicate a maximum of 4)?

- It should motivate the patient to change.
- It should change the way the patient sees things.
- It should inform about the risks of an unhealthy lifestyle.
- It should help the patient to accept the disease and its consequences.
- It should provide the patient with advice and stress management strategies.
- It should make the patient aware of her/his emotions.
- It should increase patient’s sense of self-efficacy in managing the disease.
- It should help the patient to process past traumas.
- It should help the patient to express her/his emotions and concerns.
- Other aspect(s) _____________

- In your opinion, what characteristics should the proposed physical activity have in order to promote patients’ PHYSICAL well-being?

- It should be fun.
- It should allow the interaction between the persons.
- It should be adapted to the physical needs of every patient.
- It should encourage the patient to continue with physical activity even after the intervention.
- It should have tangible benefits for the body.
- It should teach something new.
- It should let the patient get to know new persons.
- It should be a new activity.
- Other aspect(s)_______________

B07) What might be barriers for patients to participate in the meetings?

B08) Which strategies could be adopted to resolve or reduce the aforementioned barriers?

B09) What might be barriers to proposing to your patients to participate in meetings?

B10) Which strategies could be adopted to resolve or reduce the aforementioned barriers?

**SECTION C: SUGGESTIONS**

C1) If the intervention would be delivered via telematics, which benefits would you see?

C2) If the intervention would be delivered via telematics, which critical issues would you see?

**Appendix 3 Topic guide for the focus group**

The following topics will be explored during the focus groups but might be adapted later on according to the results of the survey.

- Preferences and needs in terms of psychological contents to be included in the intervention
- Preferences and needs in terms of physical contents to be included in the intervention
- Preferences and needs in terms of socio-relational aspects to be included in the intervention
- Preferences and needs in terms of intervention modalities
- Barriers to participation
- Patients’ point of view on the strategies to reduce/overcome the barriers

The following questions will guide the focus group discussions:

- Which are the psychological needs that should be targeted in an intervention?
- How should the intervention activities take place?
- With respect to physical activity, which characteristics should the intervention have?
- From a socio-relational point of view, which specific needs should an intervention meet?
- Which barriers would prevent you from participating in these intervention activities?
- Which strategies should be adopted to reduce or overcome these barriers?

**Appendix 4. Feasibility questionnaire**

**Pleasantness**

How pleasant was the intervention overall for you?

*Not at all* 1 2 3 4 5 6 7 8 9 10 *Very much*

| **What did you like most?** | **What did you like least?** |
| --- | --- |
|  |  |

**Utility**

How useful was the intervention overall for you?

*Not at all* 1 2 3 4 5 6 7 8 9 10 *Very much*

| **What was most useful for you?** | **What was less useful for you?** |
| --- | --- |
|  |  |

**Feasibility**

In your opinion, how feasible is the intervention overall?

*Not at all* 1 2 3 4 5 6 7 8 9 10 *Very much*

| **What motivated and facilitated your participation?** | **What hindered or made your participation difficult?** |
| --- | --- |
|  |  |

**Opportunities for future developments**

- How could this intervention be made more pleasant? _________________________________________________________________________________________________________________________________________________________________________________________________________________________________
- How could this intervention be made more useful/effective?

_________________________________________________________________________________________________________________________________________________________________________________________________________________________________

- What could increase the participation of other people in this intervention?

_________________________________________________________________________________________________________________________________________________________________________________________________________________________________

**Risks for future development**

- In your opinion, what difficulties may hinder other people with MS to participate? _________________________________________________________________________________________________________________________________________________________________________________________________________________________________
- What actions could encourage other people with MS to participate?

______________________________________________________________________________________________________________________________________________________________________________________________________________________
